# Cognitive Impairment in Parkinson’s Disease: An Updated Overview Focusing on Emerging Pharmaceutical Treatment Approaches

**DOI:** 10.3390/medicina59101756

**Published:** 2023-10-01

**Authors:** Yildiz Degirmenci, Efthalia Angelopoulou, Vasiliki Epameinondas Georgakopoulou, Anastasia Bougea

**Affiliations:** 1 Department of Neurology, School of Medicine, Istanbul Health and Technology University, 34093 Istanbul, Turkey; ydegir@gmail.com; 2 Parkinson’s Disease and Movement Disorders Unit, Neurology Clinic, Sisli Kolan International Hospital, 34384 Istanbul, Turkey; 3 1st Department of Neurology, Medical School, National and Kapodistrian University of Athens, Eginition Hospital, 11528 Athens, Greece; angelthal@med.uoa.gr; 4 COVID-19 Unit, Department of Infectious Diseases, Laiko General Hospital, 11527 Athens, Greece; vaso_georgakopoulou@hotmail.com

**Keywords:** Parkinson’s disease, cognition, dementia, cognitive decline, cognitive impairment, pharmacological treatments

## Abstract

Cognitive impairment in patients with Parkinson’s disease (PD) is one of the commonest and most disabling non-motor manifestations during the course of the disease. The clinical spectrum of PD-related cognitive impairment includes subjective cognitive decline (SCD), mild cognitive impairment (MCI) and PD dementia (PDD). As the disease progresses, cognitive decline creates a significant burden for the family members and/or caregivers of patients with PD, and has a great impact on quality of life. Current pharmacological treatments have demonstrated partial efficacy and failed to halt disease progression, and novel, effective, and safe therapeutic strategies are required. Accumulating preclinical and clinical evidence shows that several agents may provide beneficial effects on patients with PD and cognitive impairment, including ceftriaxone, ambroxol, intranasal insulin, nilotinib, atomoxetine, mevidalen, blarcamesine, prasinezumab, SYN120, ENT-01, NYX-458, GRF6021, fosgonimeton, INT-777, Neuropeptide S, silibinin, osmotin, cordycepin, huperzine A, fibroblast growth factor 21, Poloxamer 188, ginsenoside Rb1, thioredoxin-1, tangeretin, istradefylline and Eugenia uniflora. Potential underlying mechanisms include the inhibition of a-synuclein aggregation, the improvement of mitochondrial function, the regulation of synaptic plasticity, an impact on the gut–brain axis, the modulation of neuroinflammation and the upregulation of neurotrophic factors, as well as cholinergic, dopaminergic, serotoninergic and norepinephrine neurotransmission. In this updated overview, we aim to cover the clinical aspects of the spectrum of PD-related cognitive impairment and discuss recent evidence on emerging treatment approaches that are under investigation at a preclinical and clinical level. Finally, we aim to provide additional insights and propose new ideas for investigation that may be feasible and effective for the spectrum of PD-related cognitive impairment.

## 1. Introduction

Parkinson’s disease (PD) is a progressive and disabling neurodegenerative disorder, characterized by both motor and non-motor manifestations during the course of the disease [1]. Non-motor symptoms occur as the initial presentation in about 2% of patients with PD (pwPD) [2]. The wide spectrum of non-motor symptomatology in pwPD includes sensory disturbances and pain, sleep problems and autonomic system disorders, as well as mental disorders, including psychiatric manifestations such as psychotic symptoms, depression and cognitive impairment [2,3].

Cognitive impairment is one of the most debilitating non-motor symptoms of PD and it has a great impact on patients’ quality of life, creates a major burden on family members and caregivers, and is associated with shorter survival [4,5]. PD-related cognitive dysfunction increases the risk of institutional placement of pwPD, creating an additional economic burden on healthcare systems [6].

It has been estimated that the incidence of dementia in pwPD is about 10% per year [5].

Neuropathologically, cognitive impairment associated with PD is mainly characterized by Lewy bodies consisting of a-synuclein, as well as amyloid beta deposition, neurofibrillary tangles, microvascular damage and argyrophilic inclusions [5]. The extent of cortical Lewy body pathology in the cortex has been shown to primarily correlate with dementia [7]. The underlying pathophysiology is not clearly understood, but it is considered to primarily involve the impaired dopaminergic circuits in the basal ganglia, and the disruption of cholinergic neurotransmission in the forebrain [6]. Oxidative stress, a-synuclein and amyloid beta accumulation, neuroinflammation and mitochondrial dysfunction are among the cellular mechanisms implicated in the development of PD-related cognitive impairment [8].

Several risk factors have been shown to be associated with cognitive impairment in pwPD, including male sex and mood disorders, as well as comorbidities such as Alzheimer’s disease (AD), REM sleep behavior disorder (RBD), hypertension, diabetes and cardiovascular diseases, hyperuricemia and labile blood pressure [9,10,11,12,13]. Environmental factors like trauma and pesticide exposure, as well as genetic factors such as Apolipoprotein E (APOE) ε4, microtubule-associated protein tau (MAPT) H1/H1, Glucocerebrosidase gene (GBA1) mutations and the G196A (Val66Met) polymorphism of the *BDNF* gene contribute to its development [9,10,11,12,13]. GBA mutations are also related to an earlier onset of PD-related dementia [14], and APOE ε4, catechol-O-methyl transferase (COMT) Met/Met genotypes and the rs356219-GGpolymorphism of the *a-synunclein (SNCA)* gene have also been associated with faster cognitive decline in pwPD [6,15,16]. Alves and colleagues have also revealed an association between low cerebrospinal fluid amyloid-β 42 levels and a higher risk of cognitive decline in pwPD [17].

Cognitive impairment may occur in both early and advanced stages during the course of PD, and it is clinically characterized by considerable heterogeneity in its symptomatology. The cognitive domains affected mainly include executive and visuospatial functions, verbal fluency, processing speed and complex attention. Memory impairment may also occur during the course of the disease [18,19]. Executive dysfunction, which refers to progressive deficits in concentration, planning, organizational skills and retaining information, may appear in pwPD even at initial stages, and it often becomes worse during the progression of the disease [5]. Attention deficit is one of the most disabling features of PD-related cognitive impairment, and it is characterized by fluctuation. Visual hallucinations, autonomic dysfunction and sleep disturbances often coexist [5,19]. Although visual hallucinations may be associated with the use of anti-Parkinsonian medication, they also constitute important risk factors for the development of dementia in PD, suggesting that they are some of the core clinical features of PD-related cognitive impairment [5]. It has been proposed that the executive dysfunction associated with PD is potentially related to a hyperdopaminergic state in the prefrontal cortex of pwPD, while the progressive depositions in the posterior cortex may be associated with the irreversible damage characterizing PD-related dementia [6].

The clinical spectrum of PD-related cognitive impairment appears in a continuum of severity, and includes subjective cognitive decline (SCD), mild cognitive impairment (MCI), and Parkinson’s disease dementia (PDD) (Table 1) [18,20,21]. The neuropsychological profile of patients with PD and MCI may vary, but the most common type is single-domain non-amnestic [5]. The involvement of multiple cognitive functions has been shown to increase the risk for further decline [5]. Typically, PDD is a late feature of PD, and develops in patients with a disease duration of more than 10 years [5].

Current pharmacological treatments for PD-related cognitive impairment demonstrate partial efficacy and fail to halt disease progression. These mainly involve cholinesterase inhibitors, such as rivastigmine [5].

Accumulating preclinical and clinical evidence shows that several agents may provide beneficial effects on pwPD and cognitive impairment, including ceftriaxone, ambroxol, intranasal insulin, nilotinib, atomoxetine, blarcamesine, prasinezumab, istradefylline and Eugenia uniflora, among others, via several pathophysiological mechanisms.

Given the absence of specific disease-modifying therapies, and the important burden of PD-related cognitive impairment on patients and caregivers, novel, effective therapeutic strategies are urgently required. In this updated overview, we aim to initially cover the main aspects of the clinical spectrum of PD-related cognitive impairment and discuss recent evidence on emerging treatment approaches that are under investigation at a preclinical and clinical level, focusing on the neurobiological rationale of their potential mechanism of action. Finally, we aim to provide additional insights and propose new ideas for investigation that may be feasible and effective for PD-related cognitive decline.

It has been considered by some authors that dementia with Lewy bodies and PD dementia represent two clinicopathological conditions within the spectrum of the same disease [5]. In this review, we will focus on cognitive impairment associated with PD.

While psychotic symptoms are some of the most common non-motor manifestations of PD and core features of PDD in particular, we will include studies investigating the effectiveness of pharmaceutical treatments specifically in PD-related cognitive impairment, and not psychiatric symptoms perse. Even though cognitive impairment and psychotic symptoms share some common neuropathological and pathophysiological mechanisms, often coexist and are associated with poor quality of life, they are distinct conditions, and one may occur independently of the other [22]. Compared to PD-related psychosis, in which pimavanserin has been approved for the treatment of hallucinations and delusions, there is no specific drug approved for PD-related cognitive impairment [23].

Although our aim was not to conduct a systematic review, we followed a systematic approach for our search strategy. We searched the MEDLINE database for peer-reviewed articles written in English, investigating the effectiveness of novel pharmaceutical agents in PD-related cognitive impairment. We included both pre-clinical (in vivo) and clinical studies. In addition, we searched ClinicalTrials.gov for clinical trials investigating the effectiveness of pharmaceutical agents in PD-related cognitive impairment. No time restrictions were set, but we focused on studies during the last ten years. Studies on other non-motor manifestations of PD, such as psychotic symptoms or sleep disturbances, were excluded. In addition, studies investigating the effectiveness of cholinesterase inhibitors or the N-methyl d-aspartate (NMDA) receptor (NMDAR) antagonist memantine in PD-related cognitive decline were excluded, since these agents are already used for PDD. Furthermore, we did not include studies investigating the role of non-pharmacological treatments in PDD, such as physical exercise or deep brain stimulation (DBS). The bibliography of each relevant article was also screened to detect additional studies. Our search was performed between June 2023 and August 2023. We used the following terms, “Parkinson’s disease”, “dementia”, “cognitive impairment”, “cognitive decline”, “mild cognitive impairment”, “subjective cognitive decline”, in different combinations. After a brief summary of the clinical spectrum of PD-related dementia, our narrative synthesis was mainly based on the different pharmaceutical agents used at a clinical and preclinical level, discussing the potential neurobiological mechanisms of action.

## 2. The Clinical Spectrum of Cognitive Impairment in Parkinson’s Disease

### 2.1. Subjective Cognitive Decline (SCD)

SCD could be considered as an intermediate condition between age-related cognitive decline and MCI. In particular, it refers to a self-reported decline in cognitive function, while the neuropsychological test results are within normal limits compared to age-matched populations. These subtle, self-reported difficulties often manifest as bradyphrenia, slowed thinking and reduced processing speed in pwPD, and they may be also associated with anxiety, stress and depression [24]. The frequency of SCD in PD has not been well documented. Nevertheless, the cognitive complaints of pwPD should be regularly monitored and assessed during the course of the disease as they may eventually evolve into MCI with objective neuropsychological deficits [21,24,25].

### 2.2. Mild Cognitive Impairment (MCI)

MCI is characterized by both subjective and objective cognitive deficits with relatively preserved functional ability regarding the activities of daily living. Its prevalence ranges between 6.7 and 25.2% in the elderly population [26]. MCI prevalence among pwPD is up to 40%, and it constitutes an important risk factor for the development of PDD [20,27].

The major neuropathological characteristics of MCI in PD are Lewy body deposition in the neocortex and limbic system. However, AD-associated neuropathology has also been associated with MCI in pwPD, and it may contribute to its pathophysiology [28].

Several factors have been associated with a higher risk of MCI in pwPD, including older age, male gender, lower education status, comorbid metabolic syndrome, akinetic rigid phenotype, and the co-occurrence of non-motor features such as anxiety, autonomic dysfunction, depression and sleep behavior disorders [29,30,31].

The most appropriate neuropsychological assessment tools for PD-MCI are the Montreal Cognitive Assessment (MoCA) test, the Trail Making Test (TMT), Symbol Digit Modalities Test (SDMT), Clock Drawing Test (CDT), intersecting pentagons, Judgment of Line Orientation (JLO), Boston Naming Test (BNT), Animal Naming Test (ANT), figural memory and free and cued selective reminding tests [32].

Neuroimaging studies have demonstrated that MCI in PD is associated with cortical atrophy that involves the left prefrontal, insular, right anterior temporal, right parietal and occipital areas, and eventually the subcortical regions [33]. Moreover, hypoactivity in the occipital areas has been associated with cognitive impairment in early PD with MCI [34].

The management of pwPD with MCI involves non-pharmacological and pharmacological approaches. Physical activity and cognitive exercises have been demonstrated to be effective in enhancing global cognitive function and mental flexibility in pwPD [35,36]. However, there is currently no effective pharmacotherapy that has been clearly shown to significantly improve cognition in PD-MCI.

### 2.3. Parkinson’s Disease Dementia (PDD)

PDD is one of the most disabling and challenging non-motor symptoms, affecting about 30% of pwPD [27]. In contrast to PD-MCI, dementia cognitive deficits are severe enough to affect the activities of daily life for PD patients [7].

Older age at the disease onset and an advanced stage of PD are the most common risk factors for the development of PDD, as well as akinetic-rigid phenotype, severe motor symptoms, atypical Parkinsonian features such as symmetrical onset and early autonomic dysfunction, poor response to levodopa, male gender, low education level, vascular comorbidities such as hypertension and diabetes, RBD, presence of depression and hallucinations and genetic factors, including *GBA* gene mutations and multiplications in the α-synuclein gene (SNCA) and H1 haplotype of the MAPT gene [37,38]. Xu and colleagues have also reported that smoking history may be correlated with a two-fold increased risk of PDD [37].

The underlying pathophysiology of PDD is thought to involve deficits in the monoaminergic cholinergic and mesocortical dopaminergic system [39,40]. PDD is characterized by the degeneration of the subcortical nuclei, cortical cell death and Lewy body-type pathology, characterized byα-synuclein deposition in Lewy bodies, as well as AD-type pathology characterized by the accumulation of extracellular β-amyloid and intracellular tau protein [39,40,41].

The pharmacological treatment strategies for PDD include rivastigmine, as it has been shown to improve cognitive and behavioral symptoms [42]. Despite the insufficient evidence, other cholinesterase inhibitors including donepezil and galantamine are also considered to be “possible useful” in PDD [42,43]. Memantine, which is used for AD dementia, has also shown some benefits in PD-related cognitive decline and is also used in PDD [44].

## 3. Pharmaceutical Agents for PD-Related Cognitive Impairment: Evidence from Clinical Studies

Several drugs have been repurposed for the treatment of PD-related cognitive impairment, including PDD, and novel agents have also been developed (Table 2). In the following sections, we will provide an overview of the results of relative clinical trials investigating the effectiveness of novel therapeutic approaches in cognitive impairment in pwPD, including their possible pathophysiological mechanisms of action.

### 3.1. Ceftriaxone

Ceftriaxone, an old, third-generation, widely used cephalosporin antibiotic, has been demonstrated to exert beneficial neuroprotective effects in preclinical models of several neurological diseases [45]. Ceftriaxone can suppress glutamatergic neuronal excitotoxicity, promote the expression of glutamate transporter-1 and enhance the reuptake of glutamate. In addition, it can bind to α-synuclein and suppress its polymerization, regulate the expression of amyloid beta-related genes and improve neurogenesis [45]. Given the fact that glutamatergic excitotoxicity plays a pivotal role in the pathophysiology of PDD, it has been hypothesized that it may mitigate cognitive and behavioral deficits in animal models of PD in vivo. In this context, ceftriaxone could reverse behavioral deficits and enhance neurogenesis in 1-methyl-4-phenyl-1,2,3,6-tetrahydropyridine (MPTP)-induced rat models of PDD; in particular, it was able to improve motor function, prevent working memory and object recognition deficits, and promote neurogenesis in the hippocampal dentate gyrus and substantial nigra of the animals [46]. Interestingly, another study has indicated that ceftriaxone could exert synergistic effects with erythropoietin on the behavioral impairment and neuronal alterations of MPTP-induced rat models of PD, regarding memory deficits, as well as the degeneration of nigrostriatal dopaminergic projections and the CA1 area of the hippocampus [47]. Given these promising preclinical results, a double-blind, randomized, placebo-controlled Phase II clinical trial is investigating the safety and efficacy of the use of ceftriaxone in patients with mild and moderate PDD (NCT03413384).

### 3.2. Ambroxol

*GBA1* gene variants appear in approximately 10–15% of pwPD, and they are the most common genetic risk factors for PD development. Ambroxol has been widely used against hyaline membrane disease and airway mucus hypersecretion in newborns, and it has been demonstrated to act as a chaperone of glucocerebrosidase (GCase), a lysosomal enzyme that is implicated in the glucosylceramide metabolism [48]. Ambroxol has been demonstrated to enhance the activity of GCase and decrease GCase substrates in GBA1-mutated macrophages, and it can penetrate the brain, resulting in higher GCase levels in the cerebrospinal fluid of patients with PD carrying (or not) GBA1 mutations [48]. Although the exact underlying mechanism of its action is unclear, it is considered to involve the increased lysosomal clearance of a-synuclein [48]. Ambroxol could increase the activity of GCase and reduce the levels of α-synuclein and tau in GBA1-mutated cholinergic neurons [48], suggesting that it could exert neuroprotective effects against PDD. A phase II randomized clinical trial is investigating the effects of ambroxol on mild and moderate PDD (NCT02914366).

### 3.3. Intranasal Insulin

The repurposing of antidiabetic drugs has emerged as a novel therapeutic strategy against neurodegenerative disorders, including AD and PD. Insulin plays a crucial role in the metabolism of glucose in the central nervous system, and it displays significant neurotrophic, neuromodulatory and neuroprotective properties [49]. Intranasal insulin has been previously indicated to enhance working memory in patients with MCI and AD. Preclinical evidence has shown that intranasal insulin may also ameliorate cognitive deficits in 6-hydroxylase dopamine (6-OHDA)-induced rat models of PD by regulating the Akt/Glycogen Synthase Kinase 3 Beta (GSK3β) signaling pathway [50]. A double-blind placebo-controlled pilot clinical trial has indicated that intranasal insulin was associated with better verbal fluency among patients with PD(NCT02064166) [49]. Another randomized double-blinded placebo-controlled clinical trial has investigated the effectiveness of intranasal insulin in motor and non-motor symptoms of PD (NCT04687878), with no results published to date (https://go.drugbank.com/drugs/DB00030/clinical_trials?conditions=DBCOND0053495&phase=2&purpose=treatment&status=recruiting accessed on 26 September 2023). Further evidence is needed to clarify its efficacy in PD-related cognitive impairment.

### 3.4. Nilotinib

Discoidin domain receptors (DDRs) arereceptor tyrosine kinases which play key roles in several cellular processes, such as cell proliferation and survival. DDRs have been found in higher levels in the midbrain of pwPD. Pharmacological inhibitors of DDRs, such as nilotinib, have been shown to elevate the levels of dopamine, as well as decrease α-synuclein and hyper-phosphorylated tau in preclinical studies [51]. Nilotinib can also suppress microglia-mediated neuroinflammatory processes, thereby acting neuroprotectively against dopaminergic neuronal loss in PD models by decreasing the generation of pro-inflammatory factors such as cyclooxygenase-2 (COX-2), inducible nitric oxide synthase (iNOS), tumor necrosis factor alpha (TNF-α), interleukin-1β (IL-1β) and IL-6 [52]. Nilotinib is a potent tyrosine kinase inhibitor that has already been approved for the treatment of Philadelphia chromosome positive chronic myeloid leukemia. Given the neuroprotective potential of nilotinib in PD, its beneficial effects in PD-related cognitive impairment have also been investigated. However, an open-label study has indicated that nilotinib administration was not associated with alterations in cognition in pwPD, as assessed by the MoCA (NCT02954978) [51].

### 3.5. Atomoxetine

PDD has been shown to be associated with neuronal cell death in the locus coeruleus (LC) in post-mortem studies of pwPD [53]. LC is the major norepinephrine generator in the brain that stimulates wakefulness, and its activation has been supposed to promote attention and cognitive function [22]. Atomoxetine, a serotonin norepinephrine reuptake inhibitor approved for attention deficit hyperactivity disorder (ADHD), is hypothesized to enhance the function of the locus coeruleus. Except for norepinephrine, atomoxetine can also increase the levels of dopamine in the prefrontal cortex, since the uptake of dopamine in this brain area is mediated by norepinephrine transporters. Hence, it has been hypothesized that atomoxetine may exert beneficial effects on cognitive performance in PD, potentially by increasing the noradrenergic and dopaminergic tone in the frontal brain regions [54]. In this context, a pilot open-label clinical trial indicated that atomoxetine was effective in improving the executive deficits of pwPD without dementia [55]. In agreement with this evidence, another study demonstrated that although atomoxetine was not associated with positive effects on depressive symptoms, it could improve the global cognition of pwPD(NCT00304161) [54]. However, another randomized clinical trial in pwPD with MCI demonstrated that although atomoxetine had no significant effects on a composite score developed by a battery of executive function tests, it improved subjective measures of attention and impulsivity assessed using the Conners Adult Attention Deficit Hyperactivity Disorder Rating Scale(CAARS) [56]. This discrepancy might be explained by the potential low sensitivity of the neuropsychological tests used, combined with the possible subtle effects of the drug on cognition.

### 3.6. Mevidalen

Mevidalen (LY3154207), a selective positive allosteric modulator (PAM) of the dopamine D1 receptor, acts by enhancing the tone, affinity and response of the D1 receptor to dopamine [57]. Preclinical evidence has shown that the PAM of the dopamine D1 receptor can improve cognitive function by promoting dopaminergic neurotransmission in frontal regions, activating cortical neuronal cells, increasing synaptic neuroplasticity, and enhancing the D1-induced release of acetylcholine [58]. However, a phase II randomized, placebo-controlled clinical trial (NCT03305809) has recently demonstrated that mevidalen was not able to improve the cognitive performance of patients with mild-to-moderate dementia associated with PD or dementia with Lewy bodies (DLB) [59].

### 3.7. Blarcamesine

Sigma-1 receptors act as molecular chaperones, located at the mitochondria-associated endoplasmic reticulum membrane [60]. In preclinical models of PD, sigma-1 receptor activation has been associated with improved mitochondrial function, reduced microglial activation and decreased dopaminergic cell loss, as well as an increase in brain-derived neurotrophic factor (BDNF) and glial cell line-derived neurotrophic factor (GDNF) [60]. Blarcamesine(ANAVEX2-73) is an intracellular sigma-1 protein agonist that is being clinically investigated in a phase II randomized placebo-controlled clinical trial (NCT03774459) inpatients with PDD.

### 3.8. Prasinezumab

Prasinezumab(RO7046015/PRX002) is an anti-α-synuclein monoclonal antibody (mAb) that has shown promising results against cognitive impairment in preclinical models of PD. Prasinezumab is able to target both insoluble and soluble forms of aggregated α-synuclein [61]. Its murine parent monoclonal antibody 9E4 can ameliorate α-synuclein neuropathology and improve motor and cognitive deficits, as well as protecting against neurodegeneration in α-synuclein transgenic mouse models of PD [61]. The intravenous administration of prasinezumab has demonstrated good safety in healthy individuals [61], as well as in patients with PD [62]. A randomized, double-blind, placebo-controlled phase II clinical trial is currently investigating the efficacy of prasinezumab in PD, and the secondary outcomes include its effects on cognitive function, as evaluated by alterations in MoCA (NCT03100149).

### 3.9. SYN120

The dysregulation of serotoninergic neurotransmission is critically implicated in the pathophysiology of non-motor manifestations of PD, including depression and cognitive decline. SYN120 is a dual serotonin receptor (5-HT6/5-HT2A) antagonist that has been proposed to exert beneficial effects on the cognitive function of pwPD. However, a randomized, placebo-controlled clinical trial has recently demonstrated that SYN120 administration did not improve the cognitive performance of patients with PDD (NCT02258152) [63].

### 3.10. ENT-01

Squalamine is an antimicrobial aminosterol which has been shown to inhibit the nucleation and aggregation of a-synuclein monomers into oligomers, which display neurotoxic properties [64]. ENT-01, a synthetic salt of squalamine that has been investigated for the treatment of constipation in PD, has also been hypothesized to exert beneficial effects on cognitive function in pwPD. In this regard, a multicenter, open-label clinical trial (NCT03938922) intends to possibly resume in 2024 in order to investigate the efficacy and tolerability of ENT-01 for patients with PDD.

### 3.11. Other Agents in Clinical Trials for PDD

NYX-458 is a NMDAR modulator that improves synaptic neuroplasticity. A preclinical study has demonstrated that NYX-458 is able to improve cognitive performance in terms of working memory, attention, and executive function in primate MPTP-induced models of PD [65]. A phase II clinical trial is also investigating the effects of NYX-458 on patients with MCI or mild dementia associated with PD, as well as prodromal or manifested Lewy body dementia (NCT04148391). GRF6021 is an intravenously administered plasma-derived product, and its tolerability and safety have also been investigated in a randomized, double-blind, placebo-controlled trial for PD with cognitive impairment (NCT03713957). Fosgonimeton (ATH-1017) regulates hepatocyte growth factor (HGF)/MET, which affects neurite outgrowth and synaptogenesis [66]. This compound is also being investigated for PDD and Lewy body dementia in a randomized, placebo-controlled clinical trial (NCT04831281). Rasagiline, a selective monoamine oxidase B (MAO-B) inhibitor that increases dopaminergic neurotransmission and is currently used for motor symptoms of PD, has been shown to have beneficial effects on specific aspects of attention and executive function in pwPD without dementia in another randomized, placebo-controlled clinical trial (NCT00696215) [67]. However, another randomized clinical trial failed to reveal any significant effects on cognition in pwPD without dementia between rasagiline and placebo (NCT01382342) [68]. Another randomized clinical trial demonstrated that switching from pramipexole or ropinirole to piribedil—a non ergot dopamine agonist—had no significant effects on reaction time and the results of the neuropsychological tests of pwPD and excessive daytime sleepiness (NCT01007864) [69]. The cognitive effects of SAGE-718, a NMDAR positive allosteric modulator, have also been investigated in another two clinical trials involving pwPD (NCT05318937, NCT04476017). Furthermore, the effects of CST-2032 and CST-107 have also been investigated in patients with MCI or mild dementia due to PD or AD in a randomized, placebo-controlled, double-blind, crossover clinical trial (NCT05104463).

## 4. Pharmaceutical Agents for PD-Related Cognitive Impairment under Investigation: Preclinical Evidence

Emerging preclinical evidence has revealed the therapeutic potential of several novel candidates for the treatment of cognitive impairment in PD. Herein, we provide an overview of recent in vivo evidence regarding the effects and the neurobiological rationale for their mechanisms of action in PD-related cognitive decline.

### 4.1. INT-777

Gut microbiota and bile acid metabolism have been indicated to play important roles in PD and PD-related cognitive impairment [70]. In this context, a recent study has shown that INT-777, a 6α-ethyl-23(S)-methyl derivative of cholic acid (S-EMCA), which acts as a Takeda G protein-coupled receptor-5 (TGR5) agonist, could exert neuroprotective properties in MPTP-induced mouse models of PD in terms of cognitive and motor deficits. The underlying mechanisms were demonstrated to involve, at least partially, the regulation of neuroinflammation and mitochondrial function in microglia [71].

### 4.2. Neuropeptide S

Neuropeptide S (NPS) and NPS receptor (NPSR) have been crucially implicated in PD pathophysiology. More specifically, NPS can enhance the release of dopamine, inhibit oxidative damage, and suppress the dopaminergic neuronal loss in preclinical animal models of PD [72]. NPS has been also shown to improve memory function in MPTP-induced mouse models of PD [73], suggesting its promising potential for PD-related cognitive impairment.

### 4.3. BDNF Overexpression via AAV

BDNF is a neurotrophic factor critically involved in the molecular pathogenesis of neurodegenerative diseases, including AD and PD. A recent study indicated that BDNF overexpression via adeno-associated viruses (AAV) with BDNF gene injection was associated with an improved cognitive performance in MPTP-induced mouse models of PD, which was accompanied by a restoration of mitochondrial function and the inhibition of dopaminergic neuronal loss [74].

### 4.4. Silibinin

Another study has demonstrated that silibinin, a flavonoid derived from milk thistle (*Silybum marianum*) with hepatoprotective, antioxidative and neuroprotective properties, was able to attenuate cognitive deficits in MPTP-induced mouse models of PD. These effects were associated with reduced cellular apoptosis and a-synuclein aggregation in the hippocampus, as well as decreased oxidative stress and improved mitochondrial function [75]. Hence, this agent represents another potential therapeutic candidate against PDD, which deserves further investigation.

### 4.5. Probiotics

The oral consumption of probiotics has been associated with improved motor and non-motor symptoms in PD, such as constipation, depression and anxiety, via their implication in gut–brain axis regulation [76]. Interestingly, the administration of the probiotic *Bifidobacterium breve* could restore the abnormal synaptic plasticity in the hippocampus and facilitate fear extinction in MPTP-induced mouse models of PD [77]. In addition, the administration of the probiotic formulation SLAB51 could improve behavioral deficits and prevent dopaminergic neuronal loss in the substantia nigra pars compacta and striatum of 6-hydroxydopamine (6-OHDA)-induced mouse models of PD [78], further supporting the role of probiotics in attenuating cognitive deficits in PD.

### 4.6. Osmotin

Another agent, osmotin, an adiponectin homolog that modulates the phosphorylation of 5′ adenosine monophosphate-activated protein kinase (AMPK) through the adiponectin receptor 1 (AdipoR1), has been found to exert neuroprotective properties in preclinical models of PD. In particular, osmotin treatment was associated with better cognitive performance in a-synuclein transgenic and MPTP-induced mouse models of PD. The underlying mechanisms might involve the inhibition of α-synuclein accumulation via the upregulation of the AMPK/mammalian target of the rapamycin (mTOR) signaling pathway and the modulation of autophagy, as well as the regulation of neuroinflammation through its implication in the mitogen-activated protein kinase (MAPK) pathway [79].

### 4.7. Cordycepin

Cordycepin, a small molecule derived from cordyceps sinensis, is also able to regulate neuroinflammation in MPTP-induced models of PD by downregulating the Toll-like receptor (TLR)/nuclear factor kappa light chain enhancer of the activated B cells (NF-κB) pathway [80]. It has been demonstrated that cordycepin might exert beneficial effects on cognitive function in MPTP-induced models of PD by modulating the adenosine A2A receptors and reversing the suppression of synaptic neurotransmission in the hippocampus [81].

### 4.8. Huperzine A

The administration of huperzine A, a plant-derived lycopodium alkaloid acting as a natural acetylcholinesterase inhibitor, improved the memory and learning ability of MPTP-induced murine models of PD, which was accompanied by the prevention of dopaminergic degeneration and the modulation of inflammatory and apoptotic mechanisms [82].

### 4.9. Fibroblast Growth Factor 21

Fibroblast growth factor 21 (FGF21) displays several biological properties, such as anti-oxidant, anti-inflammatory and anti-apoptotic effects. FGF21 could prevent dopaminergic neuronal loss in the substantia nigra pars compacta, improve mitochondrial function and inhibit microglia activation in MPTP-induced mouse models of PD. Potential underlying mechanisms involved the upregulation of the AMPK/Peroxisome proliferator-activated receptor-gamma coactivator 1 alpha (PGC-1α) pathway [83]. A recent study revealed that FGF21 treatment was associated with the better motor and cognitive performance of MPTP-induced mouse models of PD, possibly by re-structuring the profile of gut microbiota, thus preventing PD-related metabolic alterations in the gut [84].

### 4.10. Poloxamer 188

It has been indicated that Poloxamer 188, an amphipathic synthetic polymer, may protect against MPTP-induced dopaminergic neuronal loss. Recently, this agent was also shown to exert beneficial effects against cognitive deficits in maneb- and paraquat-induced mouse models of PD, potentially by suppressing inflammatory responses and microglia activation and restoring hippocampal synaptic density [85].

### 4.11. Ginsenoside Rb1

Ginsenoside Rb1, the active ingredient of *Panax ginseng*, has been previously shown to attenuate motor impairment and prevent dopaminergic degeneration in MPTP-induced mouse models of PD by upregulating the glutamate transporter GLT-1 and inhibiting glutamate excitotoxicity [86]. In addition, Ginsenoside Rb1 could improve memory and spatial learning ability and enhance long-term potentiation (LTP) by upregulating the expression of postsynaptic density-95 (PSD-95) [87].

### 4.12. Thioredoxin-1 

Thioredoxin-1 (Trx-1), a redox protein, could also ameliorate memory and learning impairment in MPTP-induced mouse models of PD by regulating dopamine D1 receptor expression and modulating the NMDAR/extracellular signal-regulated kinase (ERK1/2)/cAMP-response element binding protein (CREB) signaling pathway in the hippocampus [88].

### 4.13. Tangeretin

Tangeretin, a citrus flavonoid, has also been demonstrated to suppress neurodegeneration and neuroinflammatory responses in MPTP-induced cognitive impairment of rat models of PD. In particular, tangeretin could reduce neuronal cell death in the hippocampus and the pro-inflammatory cytocines IL-1β, IL-6 and IL-2, and these effects were accompanied by improved memory function [89].

### 4.14. Istradefylline

Istradefylline, an antagonist of the adenosine A2A receptor, exerts beneficial effects on patients with advanced PD experiencing levodopa-induced motor complications by reducing OFF episodes [90]. Although the exact underlying mechanism of action is unclear, it is considered that A2A receptor antagonism may exert its effects via the modulation of gamma-aminobutyric acid (GABA) neurotransmission in the basal ganglia [90]. Notably, tradefylline administration has been associated with a better cognitive performance of MPTP-treated macaque models of PD regarding attentional deficits and working memory [91], suggesting its additional role in cognitive performance.

### 4.15. Eugenia uniflora

*Eugenia uniflora*, an extract of Brazilian purple cherry, has been associated with improved memory concerning short and long-term object recognition, social recognition and the working memory of MPTP-induced rat models of PD [92]. In this study, these behavioral effects were accompanied by the modulation of the BDNF/tropomyosin receptor kinase B (TrkB)/p75^NTR^ axis in the hippocampus, which is implicated in synaptic plasticity and neurotransmission. Hence, its clinical efficacy in PDD should be further explored in the future.

## 5. Conclusions and Future Perspectives

Cognitive impairment in pwPD is one of the most disabling and challenging non-motor manifestations during the course of the disease, and has a negative impact on patients’ quality of life. Current pharmacological treatments, mainly including cholinesterase inhibitors, show only partial efficacy, and fail to halt disease progression. A growing body of preclinical and clinical evidence has investigated the therapeutic potential of several repurposed and novel agents against PD-related cognitive impairment. Potential underlying mechanisms of action include the inhibition of a-synuclein aggregation, the improvement of mitochondrial function, the regulation of synaptic plasticity, the impact on the gut–brain axis, the modulation of neuroinflammation and the upregulation of neurotrophic factors, as well as cholinergic, dopaminergic, serotoninergic and norepinephrine neurotransmission. However, despite the promising evidence from preclinical studies, the available results of most clinical trials are rather disappointing, showing no or only partial benefits.

The reasons for this discrepancy are probably multifactorial. Firstly, the exact etiology of the dopaminergic neuronal cell death and the neurodegenerative process of PD and PD-related cognitive impairment is still unclear. Although some genetic and environmental factors and other comorbidities, as well as disease characteristics, have been associated with the development of PDD, the underlying pathophysiological mechanisms with the most significant contribution to the pathogenesis and progression of PDD remain to be elucidated. For this purpose, instead of focusing on a specific neurobiological mechanism or signaling pathway, it could be proposed for future preclinical studies that the “proportional” contribution of each factor to the development and progression of PD and PDD, in particular, is investigated. Furthermore, there is still no animal model that can effectively reflect the pathogenesis and the pattern of pathology of PD and PD-related cognitive impairment in humans [93]. The animal models used are acute and non-progressive, which do not characterize the chronic nature of PD-related cognitive impairment. The heterogeneity of PD and PDD in humans also needs to be reflected more effectively in preclinical models [94] in terms of the clinical presentation and the underlying pathophysiological mechanisms. Hence, the development of preclinical models that can more accurately reflect the PD and PDD neurobiology in humans are of paramount importance.

In addition, timing is another important factor for the investigation of neuroprotective therapies in neurodegenerative diseases, including PDD. In preclinical studies, researchers can more easily select the probable best timing for the administration of the drug, which is more difficult for clinical studies in humans. It is considered that the delivery of an intervention at earlier stages of neurodegenerative diseases is generally desirable [94]. In this context, the development and validation of biofluid or neuroimaging biomarkers able to detect or predict with high accuracy the PD-related cognitive impairment at earlier, pre-symptomatic, prodromal states would be very useful.

The clinical trials described above also have some limitations. Some studies are probably underpowered, since they have been conducted at a pilot level; hence, the potential therapeutic benefits of the relative drugs on PD-related cognitive impairment might have been missed. Moreover, the optimal dose of each drug is sometimes difficult to determine, especially in cases where a neuroprotective effect is anticipated [93]. The selection of an appropriate outcome measure is also a crucial factor [95]. Most studies have used alterations in neuropsychological tests as outcome measures, such as MoCA, or scores of tests investigating other domain-specific instruments, such as executive function or attention. Given the fluctuating clinical features of PDD, it can be hypothesized that the performance in these tests might not precisely reflect the progression of the underlying neurodegenerative process of PDD. Hence, the development and the use of appropriate and neuroimaging serum or CSF biomarkers that could accurately reflect the disease stage would be very useful in relative clinical trials. Although no biomarker for PDD has been validated yet, several studies have demonstrated that reduced levels of epidermal and insulin-like growth factors and uric acid in the serum/plasma, low levels of amyloid beta in the CSF, hippocampal atrophy in MRI and decreased cholinergic innervation and metabolism in the brain, as assessed by PET, primarily in posterior regions, might be associated with PDD, paving the way for their potential use in clinical trials [96].

As PD-related cognitive impairment appears on a clinical spectrum, the inclusion of pwPD with various forms of severity of cognitive complains and deficits (normal cognition, SCD, MCI, PDD) would probably affect the results of the clinical trials. It also remains unclear whether patients with PDD are better combined with patients with dementia or with Lewy bodies in clinical trials [57].

Given the considerable clinical heterogeneity of PD and PDD, it is likely that partially different molecular mechanisms might underscore the neurodegenerative process in patients with PD-related cognitive impairment. The identification of a “molecular signature” for each patient could aid in the development of effective and personalized treatment strategies. The inclusion of biomarker-defined sub-populations of patients with PDD, beyond just using the clinical diagnostic criteria, could represent a more appropriate approach. Although this strategy may be associated with important logistical and financial challenges [94], the growing use of telemedicine in clinical trials for the increased recruitment and closer follow-up of participants represents an attractive solution [97].

Future clinical trials could involve patients with PDD carrying mutations in genes associated with cognitive impairment, such as GBA or SNCA. This approach can provide a significant opportunity to understand the pathophysiology of PDD in more depth and develop targeted pharmaceutical approaches. For instance, ambroxol, which increases GCase activity and promotes autophagy-lysosome degradation molecular pathways, could be specifically investigated in carriers of GBA1 mutations and PD-related cognitive impairment. In addition, the efficacy of intravenous infusions of monoclonal anti-α-synuclein antibodies, which have been used in clinical trials for PD [61], could be investigated in the case of PD-related cognitive impairment in pwPD with SNCA gene mutations.

In the same vein, given the high heterogeneity of the progression of PD-related cognitive impairment, efforts should be made for the inclusion of patients with similar progression rates. In this context, in a clinical trial of azathioprine in pwPD, a prognostic risk score was developed and only individuals with a higher risk of progression were included in the study [98].

Given the promising potential of non-pharmacological treatments in PD-related cognitive impairment, it can be also hypothesized that the investigation of combined pharmacological and non-pharmacological therapeutic approaches, such as physical exercise or DBS, may constitute the best approach [57].

The relatively low recruitment of clinical trials in PD is another critical factor that should be considered, because of the fear of possible side effects, the interruption of the ongoing patients’ medical regimen and concerns about receiving placebos. In this regard, effective communication between physicians and participants in order to educate the patients and address potential misconceptions concerning the clinical trials could stimulate the engagement of patients with PD in research [95].

In conclusion, PD-related cognitive impairment represents a devastating non-motor manifestation of PD, with no disease-modifying therapeutic option available to date. Despite the extensive research and the promising preclinical evidence, clinical trials have shown partial or no benefits of the investigating drugs. A better understanding of the underlying pathophysiology of PD-related cognitive impairment, and an improved methodology for preclinical and clinical studies, are needed in order to develop effective disease-modifying pharmaceutical therapies.

## Figures and Tables

**Table 1:** Characteristics of the clinical spectrum of cognitive impairment in Parkinson’s disease.

Characteristic	PD-NC	PD-SCD	PD-MCI	PDD
Subjective complains	No	Yes	Yes	Yes
Objective deficits in neuropsychological testing	No	No	Yes	Yes
Cognitive deficits	No	No	Most commonly executive dysfunction	Executive dysfunction, visuospatial deficits, memory impairment, visual hallucinations, attention deficits, fluctuations
Functional impairment in ADLs	No	No	Relatively preserved	Yes

PD-NC: Parkinson’s disease—normal cognition; PD-SCD: Parkinson’s disease—subjective cognitive decline; PD-MCI: Parkinson’s disease—mild cognitive impairment; PDD: Parkinson’s disease dementia; ADLs: activities of daily living.

**Table 2:** Investigating drugs for cognitive impairment in Parkinson’s disease and their current status.

Investigating Drug	Clinical Trial Number	Clinical Trial Status (Date Accessed ClinicalTrials.gov: 16 September 2023)
Ceftriaxone	NCT03413384	Ongoing
Ambroxol	NCT02914366	Ongoing
Intranasal insulin	NCT02064166, NCT04687878	Completed, Unknown
Nilotinib	NCT02954978	Unknown
Atomoxetine	NCT00304161	Completed
Mevidalen	NCT03305809	Completed
Blarcamesine	NCT03774459	Completed
Prasinezumab	NCT03100149	Ongoing
SYN120	NCT02258152	Completed
ENT-01	NCT03938922	Suspended
NYX-458	NCT04148391	Ongoing
GRF6021	NCT03713957	Completed
Fosgonimeton	NCT04831281	Ongoing
Rasagiline	NCT00696215, NCT01382342	Unknown, Completed
Piribedil	NCT01007864	Completed
SAGE-718	NCT05318937, NCT04476017	Ongoing, Completed
CST-2032 and CST-107	NCT05104463	Ongoing
